# The Intersection of Workplace and Environmental Exposure on Health in Latinx Farm Working Communities in Rural Inland Southern California

**DOI:** 10.3390/ijerph191912940

**Published:** 2022-10-10

**Authors:** Ann Marie Cheney, Tatiana Barrera, Katheryn Rodriguez, Ana María Jaramillo López

**Affiliations:** 1 Department of Social Medicine Population and Public Health, School of Medicine, University of California Riverside, Riverside, CA 92521, USA; 2 School of Medicine Undergraduate Medical Education, University of California Riverside, Riverside, CA 92521, USA; 3 Department of Anthropology, College of Humanities and Social Sciences, University of California Riverside, Riverside, CA 92521, USA; 4 Estudios de Población, El Colegio de la Frontera Norte, Tijuana 22560, Baja California, Mexico; 5 Comisión de Salud Fronteriza México—Estados Unidos, Tijuana 22010, Baja California, Mexico

**Keywords:** farmworkers, Latinx, foreign-born, immigrant, Mexican, Salton Sea

## Abstract

Workplace and environmental exposures pose health risks for racial/ethnic minorities in rural agricultural communities, placing them at a disadvantage in accessing needed health care. Over three fourths (76%) of the 2.4 million farmworkers in the United States are immigrants, mostly from Mexico. However, little is known of the community health concerns and barriers to care of Latinx farmworkers in inland southern California. This qualitative study used a community-based participatory research approach, conducting nine in-home meetings to obtain meaningful community input on health concerns and barriers to access healthcare services among rural residents of the Eastern Coachella Valley, who are also located near the desert-bound Salton Sea of inland southern California. All interviews were audio-recorded and analyzed via listening to the audio recordings and summarizing data in templates and matrices. Participants discussed health concerns related to agricultural labor, including heat-related illness, musculoskeletal ailments and injuries, skin disorders, respiratory illness, and trauma. Participants raised concerns about environmental exposures related to agriculture and the nearby Salton Sea, a highly saline lakebed, and proposed solutions to improve the health of their communities. The findings from this study suggest farmworkers are aware of the health risks posed by living and working in rural farmlands but lack resources and information to act upon and advocate for improved public health.

## 1. Introduction

US agricultural production has doubled since the beginning of agribusiness in the 1950s, with a decline in family-run farms and US-hired farm workers and an increase in the employment of contract-based foreign laborers. Over three fourths (76%) of the 2.4 million farmworkers in the US are immigrants, most of whom are from Mexico and authorized to work in the US (NAWS 2015–2016) [1]. In comparison to other Latino/Hispanic ethnic groups, Mexican immigrants are much more likely to work as farm workers. Factors such as undocumented status, limited education (i.e., 57% have not completed high school or an equivalent), and stereotypes associated with the “Mexican worker” contribute to the high concentration of Mexican immigrants in low-wage, contingent labor such as farm labor [2].

Because of the seasonal nature of farm work, laborers typically do not work year-round: most average 33 weeks of employment/year at an annual income well below the poverty line (a household of four earns ~$17,500/year; US poverty guideline is $26,200/year). Over a third of the foreign-born farmworker population has lower than a sixth-grade education and the vast majority speak Spanish as their primary language (NAWS 2015–2016). Furthermore, this population of farmworkers often live in crowded dwellings, substandard housing, and may live where they work [3,4,5].

Despite holding one of the most dangerous jobs in the US [6], farmworkers normally stay in the agricultural industry for years. Farm work inherently exposes laborers to heat, cold, and ultraviolet (UV) rays, which increase their risk of injuries related to environmental exposures [7]. Pesticides, while contributing to increases in crop yields [8,9], also contribute to poor health conditions among farmworkers who have considerably higher exposure to pesticides than non-agricultural workers, increasing their risk for skin disease, vision problems, and respiratory-associated illness [8,10,11].

In addition, the picking of crops, repetitive movements, heavy lifting, and lengthy periods of standing or kneeling is associated with injuries, fractures, and chronic pain among farmworkers [12,13]. The fast-paced, high-risk working environment is stressful, which can contribute to depressive and psychiatric symptoms [14,15]. Substance abuse and sexual risk behaviors are also prevalent in the US farm worker population. Male farmworkers in particular report paying for sex, infrequent condom use, illicit drug use (including during sex), and high rates of alcohol use [13,16,17,18].

These are just some of the many illnesses and conditions linked to agricultural labor. However, illness and disease are also shaped by the local environment and geographic locale. Social and economic factors largely determine where individuals live, work, and grow and are directly associated with health outcomes [19]. In what follows, we highlight community perceptions of common health concerns among Latinx farm working communities and proposed solutions to addressing workplace and environmental factors contributing to poor health outcomes. The aim of this article is to shed light on the intersections of workplace and environmental exposures on the health and wellbeing of families and children in farm working communities.

## 2. Materials and Methods

The analysis presented in this study is part of a larger research study reporting on binational approaches and policies to address the healthcare needs and access barriers of Mexican migrants in the US [20,21]. This study intended to understand health concerns and barriers to health care among the predominantly Mexican-immigrant, farm working population in the Eastern Coachella Valley (ECV) located in inland southern California—an unincorporated rural area that, despite being categorized as desert, is among California’s top regions for agricultural production. We conducted in-home meetings, guided by trained Latinx community leaders, in participants’ native language(s). Prior to the start of research, we obtained approval from the University of California Riverside (UCR) Institutional Review Board (IRB).

*Approach:* With the goal of promoting community involvement in identifying health priorities and grassroots solutions to existing healthcare barriers for Latinx farmworkers, we applied a community-based participatory research (CBPR) approach [22]. In line with CBPR approaches, at the beginning of the project we established a community advisory board (CAB) of 12 members: five community health workers, Spanish-speaking promotoras, four of whom served as community representatives from each of the four neighborhoods of the ECV, two representatives each from local healthcare systems and community-based organizations, and an academic researcher. The CAB met monthly during the study period, reviewed, and revised all interview guide materials, provided guidance on recruitment strategies, validated the interpretation and translation of data, and assisted with the dissemination of study findings. Additionally, CAB members participated in an asset mapping activity that identified the cultural, economic, political, and health resources in each of the four communities in the ECV.

**Figure 1:**
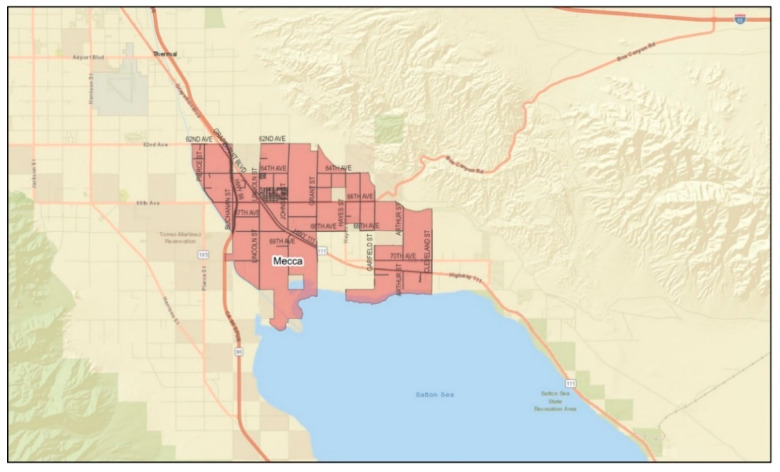
Geographical division of the community of Mecca. (Source: Riverside University Health System—Public Health).

**Figure 2:**
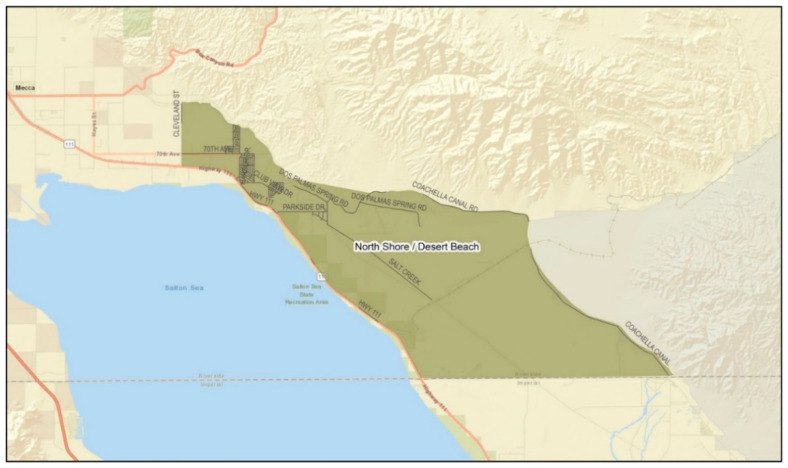
Geographical division of the community of North Shore. (Source: Riverside University Health System—Public Health).

**Figure 3:**
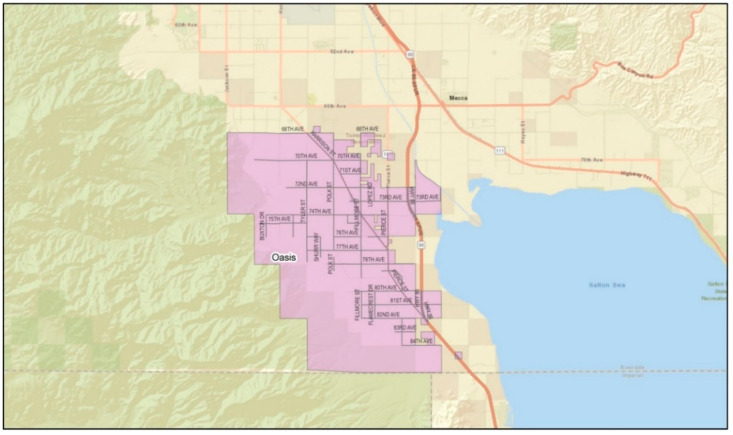
Geographical division of the community of Oasis. (Source: Riverside University Health System—Public Health).

**Figure 4:**
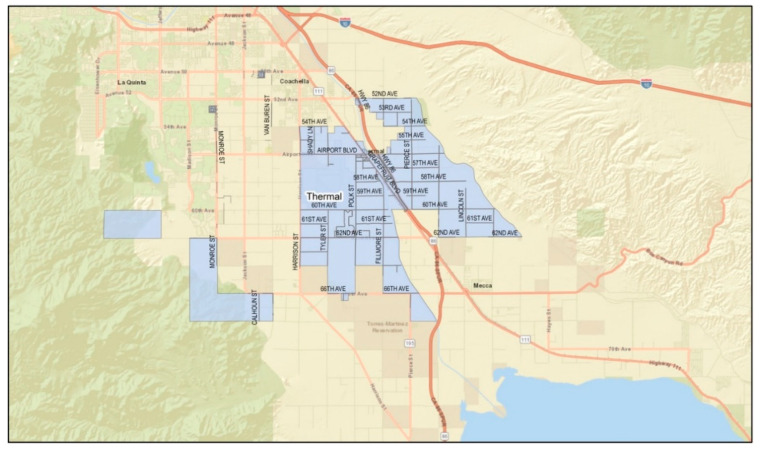
Geographical division of the community of Thermal. (Source: Riverside University Health System—Public Health).

*Setting:* California is the national leader in agricultural production [23]. Unlike other farming regions (i.e., the US Mid-West, Northeast, Northwest, and Southeast), California’s agricultural production is year-round, and over half of its farm sales come from labor-intensive crops (e.g., fruits, nuts, vegetables, and melons). The state’s warm climate also serves as one of the most unforgiving conditions for its laborers, placing them at risk of heat-related illness (HRI) [24]. The ECV, where the study was conducted, ranked 14th among agricultural counties in the state [25]. The eastern valley is a geographical subdivision consisting of four rural and unincorporated communities: Mecca, North Shore, Oasis, and Thermal (Figure 1, Figure 2, Figure 3 and Figure 4). Its more than 200,000 acres of farmland serve as a main source of employment for many, particularly for Latinx immigrants, principally of Mexican origin, living in poverty [26]. More than 6000 to 10,000 immigrants in the ECV identify their origins to an indigenous Purépecha community in the Mexican state of Michoacán [27].

Latinx farmworkers in the ECV face a number of social, economic, and environmental vulnerabilities because of their social and legal statuses and proximity to the Salton Sea [3]. The Salton Sea, a current ecological disaster, is a 345 sq. mile land-locked body of water. The boundaries of the four unincorporated communities touch the northern part of the Salton Sea. For decades, its water levels were maintained by municipal sewage, rain, and agricultural runoff [26]. The Salton Sea’s main water source today has been reduced to primarily agricultural runoff [28]; between drought and water politics, the Salton Sea’s water levels are dropping, its borders receding, and its salinity increasing—exposing emissive surfaces increasing wind-blown dust and contributing toxic air contaminants, including ammonia and sulfur dioxide gases [29,30,31]. Dust storms transport exposed playa containing toxic metals, pesticides, and other toxins into the communities and homes of those living along its borders, which may contribute to severe health conditions such as asthma and other respiratory distress [32,33].

Bounded by Southern California’s mountain ranges, the ECV’s year-round warm climate makes it conducive to agricultural cultivation; even so, high summer temperatures and drying desert winds expose farmworkers to long hours in working conditions that can include temperatures over 37 °C in the spring and summer months, with 2019 mean monthly maximum and minimum temperatures of 44.2 °C and 4.9 °C, respectively, and total monthly precipitation ranging from 0.00 to 36.3 mm [34].

Deaths due to heat exposure and dehydration are a concern in this region: since 2005, the California Division of Occupational Safety and Health (Cal-OSHA) has required employers to provide employees with training on recognition and prevention of heat-related illness as well as fresh water and shaded rest-breaks. Despite legislative efforts, recent years show no downward trend in heat-related deaths (HRDs) or illnesses (HRIs); spikes in HRDs and HRIs are seen during years with record-breaking heat-waves [34]. Illness and injury associated with workplace exposures and conditions continue to be a concern among farmworkers in the state of California, with agriculture as the industry with the highest average work-related fatality rates in the state [35,36,37,38].

*Participant Eligibility and Recruitment:* Participants were eligible if they (1) were 18 years of age or older, (2) lived in one of the four communities bordering the northern part of Salton Sea (North Shore, Mecca, Thermal, Oasis), and (3) spoke English, Spanish, and/or Purépecha. The five *promotoras* disseminated a study flyer to members of their immediate community. The flyer included information about the study, the goals of the talk, and the incentives which was a raffle prize.

*Data Collection:* From 2017 to 2018, we conducted research on community health priorities and access barriers to healthcare service use. Study team members provided participants with consent documents outlining the research, its goals, participant remuneration, risks and benefits to research participation, and IRB contact information. 

We conducted nine in-home meetings, seven exclusively in Spanish and two in Spanish and Purépecha, a language indigenous to Mexico. In-home meetings are a culturally appropriate group interview method whereby a host family invites friends, family, and neighbors to their home [39]. The lead *promotora*, a female bilingual Spanish-Purépecha Latina, and a Latino male researcher were trained to facilitate group interviews. Facilitators used a semi-structured interview guide to elicit responses and guide group discussion. Open-ended questions elicited information on community health priorities, barriers to healthcare access, and ideal models of healthcare service delivery (see Table 1 for interview guide). Upon completion of the discussion, participants were asked to fill out a brief socio-demographic survey. We provided a meal, a small gift ($3 to $5 value), and the opportunity to enter a raffle for a prize valued at $60 to $75. All host families received a $60 supermarket gift certificate. Group interviews lasted 60 to 90 min. Data collection stopped when we reached data saturation, meaning participant responses were similar resulting in new data redundancy [40].

*Data Analysis*: All interviews were audio-recorded and analyzed using rapid analytic techniques involving listening to the audio recordings and summarizing data in templates and matrices [41,42]. Study team members listened to the audio recordings from each in-home meeting and input relevant data, including quotes and themes, into templates. A matrix organized by in-home meetings (as rows) and interview questions (as columns) was used to identify patterns and key themes.

## 3. Results

### 3.1. Participant Characteristics

In total, 82 adult residents of the four unincorporated neighborhoods of the ECV attended the nine in-home meetings. Their characteristics, based on post in-home meeting surveys (*n* = 76), are summarized in Table 2. The majority were Mexican immigrants, women, low-income, and also lived in a trailer. Over a third (36%) were over 50 years of age. Nearly 60% were currently employed as farmworkers; 86% were Spanish-only speakers, 7% were Purépecha speaking only, and 7% were bilingual in English and Spanish. Of those surveyed, only 63% had a healthcare visit in the past year. Less than half (43%) of participants had health insurance.

### 3.2. Environmental Exposures

The majority of participants (59%) work in agriculture (Table 2). Many reside in close proximity to farmlands, where they are regularly exposed to pesticides, chemicals, agricultural runoff, and mosquitos. As outdoor workers, they are constantly subjected to high levels of UV and heat exposure, as well as regional wind storms that carry dust and toxins from the nearby desiccating Salton Sea.

There is a disproportionate prevalence of respiratory illness in the ECV. Approximately 20–22.4% of children living along the Salton Sea meet the diagnostic criteria for asthma or other respiratory illness [33], compared to 10% for children in California and 11.3% in the U.S. as a whole [43]. Participants at all in-home meetings raised concerns about the prevalence of respiratory illness in the ECV, linking the local decline of respiratory health to environmental exposure via the interrelation of the Salton Sea and their agricultural labor.

### 3.3. The Salton Sea

Participants were aware of the negative effects of the Salton Sea on their health, and that their constant exposure while working in the fields made matters worse. They discussed how the dust and smells of sulfur and fish in the air contributed to poor eye health and poor vision, itchy skin, nosebleeds, sinusitis, colds, allergies, and headaches. Furthermore, participants believed the Salton Sea and regional dust storms caused asthma, bronchitis, and other breathing difficulties among farmworkers and those living in the region especially children.

“At my work, you hear a lot about [disease of] the lungs, allergies, because there, well at work, lots and lots of soil blows, and that’s what gives lots of allergies or headaches.”

“Bronchitis, because that’s what you get now with those winds that come with all the dust and you start with chest pain and it’s bronchitis.”

“The [people] who are near the lake, the smells and that water…. There’s a lot of colds, allergies, sinusitis, asthma…. Supposedly, it gives asthma to children, it’s infected many with asthma…. In their noses, it [the Sea] smells awful.”

Some participants, aware of the poor air quality, inquired about the validity of the link between the Salton Sea and disease, suggesting they lacked information on the health-related risks associated with the Salton Sea.

“I have a question, regarding the diseases of the [Salton Sea], how true is it that the [Salton Sea] produces … like right now that you can feel that the air is the kind that can damage the lungs of the children, how true is that?”

### 3.4. Agricultural Labor Impacts on Health

In all in-home meetings participants discussed the effects of extreme heat conditions, long work hours, bending and lifting, excessive consumption of caffeine, little water consumption, and pesticide exposure on chronic health conditions and psychological and emotional distress. They were aware that their status as immigrants (documented or undocumented) exposed them to these negative labor conditions:

“When have you seen [Americans] working their tail off, in this blazing heat, getting sunburnt, with thorns? I work in the date [farm], I am all pricked, all blistered. When do you see *a gabacho* [White person] all pricked? Or scratching and scratching? They aren’t like us [immigrants], working ourselves to death out in the field. On our knees.”

They also noted some health conditions were directly connected to workplace negligence and their inability to ask for better work conditions. Participants raised concerns about employers failing to provide cool water and workplace training on heat-related illness. Participants said the water provided was often hot due to the high temperatures outdoors. Having to work under extreme heat without appropriate hydration, they explained, put farmworkers at risk for heat exhaustion and other heat-related illness—topics about which they lacked information. Participants also stated farmworkers are afraid to speak up to superiors about workplace conditions.

“Because of the [high] temperatures, you can get heat exhaustion, but you don’t inform [a supervisor]. You can deal with it because they’re going to fire you or because you need work. But you must speak up… And they don’t give you information. They only give you a workplace training one time, but you need to be informed.”

*Chronic health conditions.* Participants extensively discussed the impact agricultural labor has on their physical health, ranging from headaches to hearing loss from noisy machinery to musculoskeletal conditions (e.g., foot and knee injuries from standing or kneeling for extensive hours; pain, swelling, heel spurs, knee effusions, trouble walking, etc.), back and sciatic nerve pain from heavy lifting, deviated discs, muscle sprains, and fractures. They also discussed skin conditions related to workplace exposures (e.g., itchiness, rashes, eczema, warts from sharing gloves at work, skin allergies, and skin cancers). Although the consensus was that working long hours in the heat and under the sun’s rays was a major contributor to chronic health conditions, participants agreed that exposure to dust, soil, pesticides and chemicals used on the fields either caused or exacerbated these skin conditions and contributed to the respiratory and vision problems they and their families experienced (e.g., asthma, nasal allergies, infections, bronchitis, burning eyes, etc.).

Participants were also aware of the threat posed by pesticides and chemicals—so much so, they believed pregnant women should not work in the field. Autism, ADHD, learning disabilities, developmental or genetic disorders, Down Syndrome, and birth defects were discussed as conditions linked to chemical exposure.

“I think that many children are born that way with mental delays, possibly because the dad used drugs, or the mom, or because of the chemicals, or because of what they spray here over the plants and field. Because I’ve seen a lot of kids here who have mental delays.”

“The ones who are pregnant, the chemicals they spray on the vegetables and all, that’s why they come out sick, the kids, the babies. They are [born] sick, too, because of that. The pesticides.”

The attendees also mentioned kidney disease, prostate cancer, and other deaths in their communities they attributed to pesticide and chemical exposure.

“There are many people that suffer from the chemicals. We have a family member who died from that. He breathed in too many chemicals and died. Intoxication of chemicals. And that’s [also] a cause of prostate cancer.”

*Stress and trauma of labor conditions.* In addition to chronic physical health conditions, farmworkers also experienced stress and trauma connected to their work conditions. Participants connected physical exhaustion from work with mental exhaustion. Working mothers stated insomnia and/or lack of sleep due to waking up at dawn to go to work and having long hours of physical labor, in addition to household chores and duties, which caused stress and worsened physical ailments. One participant shared how they lived with trauma and jitteriness after having accidentally run over a coworker with machinery.

“To be honest, I do suffer a bit from nervousness… I think that maybe I did end up traumatized. Like scared. [My employer] told me to go see a psychologist so that I could receive therapy, but I felt fine. But, like, sometimes I don’t know, I just jump. I’ll be sleeping, and suddenly I jump.”

Participants acknowledged the importance of treating mental health in a timely manner to prevent suicide. They believed depression and high stress levels could lead to physical illness, heart attacks and strokes, but that such mental illnesses went undiagnosed or untreated. Some expressed frustration: they wanted to share their mental health struggles but feared the result of seeking care, either due to being immigrants and/or being identified as a threat to oneself or others:

“I, [have depression], because I lost a daughter… It’s just that here, when a person is depressed, it’s a threat because they’re supposedly suicidal, they are going to kill themselves or harm someone, but it’s not like that; if it’s treated in time, it’s not like that… Many people, because they’re in this country, I’m referring to Latinos, we don’t go to a doctor. We [can’t] say, ‘Oh, yes, I feel depressed, I cry a lot, I feel a void, I feel very lonely…’ because then they tell you, ‘Have you tried to commit suicide? Who have you hurt?’ And it’s not like that. I don’t want to kill myself; I am just sad.”

Seeking psychological help is not common practice in immigrant Latinx communities, especially among men, but the additional burden of losing time at work to travel to distant sources to obtain mental health care made it inconvenient. Others, who were not uninsured, could not afford to seek psychological care. However, access and cost were not the only limiting factors. At all in-home meetings, participants wanted health information, including on mental health, and they shared the importance of training doctors on the signs of mental illness. Participants suggested mental health surveys were needed to better identify psychological conditions.

*Coping with labor conditions.* Others talked about the physical pain of their labor conditions and common practices used to deal with such pain. Use of pain medications, including codeine and prescription painkillers, is a widespread practice in these communities. Because the majority of immigrants in the ECV are uninsured, self-medicating (through alcohol, illicit drugs, etc.) and sharing medications is commonplace, as is obtaining medications from Mexicali, Mexico where doctor’s visits and medications are more affordable and sometimes of better quality.

“There are many people medicated or self-medicated… It’s like a bit of an irony that mom and dad are against [teens using drugs], but they are also really drugged at the workplace.”

Some participants were unaware of self-medication with pain killers in the workplace, until they experienced it first-hand. For instance, one participant shared:

“One day I had a headache and, I asked the supervisor, ‘Do you have any pills for headaches?’ and she told me ‘No, but I have, here have some of this.’ And she gave me one [opioid], and boy did they have me high. She bought them in Mexicali. I felt like a drug addict, like I was flying. They’re the ones they call opioids, but she said they were the prescribed kind and that’s why she’d get them from Mexicali. They don’t sell them here unless you have a prescription. That’s why she gets them there.”

Other participants said they gave their painkillers and prescription medications to co-workers. They stated many in their community do not have access to treatment options and medications due to cost, no local pharmacy, and the lack of health insurance coverage.

Participants also discussed drinking multiple caffeinated beverages (e.g., energy drinks such as Red Bull and Monster) and mixing painkillers with energy drinks as a common practice used to stay alert and meet work demands. Although some participants were aware of the consequences of energy drinks (i.e., kidney disease, stroke, diabetes due to the high sugar content, and addiction to caffeine), they stated that the need to make a living and keep their jobs was more important than the possible negative health effects. One participant commented: “You can’t work if you don’t drink Monster.”

This fear of losing their jobs creates a situation where many farmworkers stay quiet when it comes to unsafe workplace conditions and injuries. Participants spoke of times when they noticed health risk factors in the workplace (e.g., the lack of clean restrooms, cool water, and workplace trainings noted above), but did not voice their concern out of fear of being fired and thereby losing the ability to provide for their families. Similarly, many do not report workplace injuries out of fear, representative of their lack of information regarding their rights as farmworkers.

### 3.5. Community Proposed Solutions

In response to the harmful environmental exposures and noxious air, participants stated a need for community involvement, improved sewage systems, and increased pest control, as well as clear information on the environmental health hazards of the ECV and Salton Sea.

As an example, participants said they had been informed via the public radio about a recent outbreak of mosquito-borne disease in a neighboring community and suggested aerial insecticide spraying might help, but also accepted that, given they live in close proximity to the fields and work in them, exposure to mosquitoes was inevitable.

“There are many mosquitos here, and more so here because you know there are a lot of puddles… A lot of water is thrown below the plants, they need water to grow, and that is where mosquitos breed, and a lot of mosquitos.”

Participants understood that their respiratory illnesses were a chronic and growing problem related to their work and environment, and also that they could better manage their health with the right tools. One participant suggested local classes, similar to diabetic health coach-led classes, on respiratory illness would be helpful.

“…And also to take a class on what is bronchitis. I think there are classes on breathing [problems], because there are breathing classes for people who have that problem.”

In addition to respiratory illness, local health-coach led classes free to the public could inform ECV residents of other health-related topics (e.g., diabetes, cholesterol, weight management, healthy eating, STIs and reproductive health, and workplace rights). Ideally, a variety of classes would be open to the community to improve health quality. Arts and crafts classes for adults and youth could help relieve stress and promote mental wellbeing. In addition to constructing outdoor recreational options (e.g., parks), air-conditioned gyms could provide farmworkers and their families the option to pursue recreational activities in a comfortable and safe environment. This, coupled with an instructor to show people living with chronic health conditions or work-related injuries how to appropriately exercise and eat well, could prove even more effective than medications, participants said. They also stated their belief that local health workshops and group discussions could foster a sense among community residents and farmworkers that one’s health should be a priority.

At every in-home meeting, participants brought up their need for more health-related information. Although a few participants were uncertain about commenting on community health conditions because they knew little about medical topics, many made accurate associations between cholesterol and heart disease, HPV and cervical cancer, and sun exposure and skin cancer, etc. Participants acknowledged the relationship between their lack of information and a lack of confidence to act upon their health: they either did not seek treatment for a health concern or failed to do so before it turned into a chronic illness or fatal disease.

“You can also get cancer from being in the sun. In the skin, you get [cancer], but [cancer] can later be in the blood. When a person has leukemia and you work in the field, they don’t know they have leukemia. You get [cancer]—but most people don’t know when they have leukemia. They just say, ‘Oh I think I have that,’ but they don’t go get checked… Also, many people don’t know what cancer is, because there are many forms of cancer you can have. I’ve seen it on TV.”

Participants asked which illnesses they needed to be aware of and their causes, symptoms, treatment, and prevention. Were they to have the right information, they would use it to not only better their own health, but also that of their families.

“Anemia … one doesn’t know what anemia is, sometimes one doesn’t know. They don’t inform us. [People] just say, ‘Oh, my kid got anemia,’ but [they] are not informed… But when one doesn’t know, one doesn’t take heed. That’s why more information is so important.”

With the information in hand, they stated the stigma associated with having an illness or visiting a doctor could be minimized; the shame of being identified as ill, overcome; and the fear of losing their jobs, dissipated.

“… Many people who are injured at work do not speak up, so long as they don’t get fired. Because you don’t have information, you have to obtain information regarding injuries: that you have rights to speak up over why you feel sick and that they should pay for the days that you didn’t work. But no, we go to work saying, ‘No, they will fire me’. But no, you have to have the right information.”

Across all discussions, participants discussed the need for structural neighborhood and workplace changes as essential for the improvement of their communities’ health. Participants wanted improved road and sidewalk security (e.g., via more lighting, policing, etc.); expanded bus stops and routes for those without a vehicle to drive to medical appointments; recreational parks where children and adults could relieve stress and stay healthy; neighborhood daycare centers to care for children while they attended their medical appointments, and a greater availability of supermarkets (many participants only had one local supermarket, with prohibitive prices). Some participants suggested acquiring signatures to advocate for the much-needed changes. One participant expressed the need for an advocate, especially when it came to addressing workplace conditions.

## 4. Discussion

With restrictions to employment including language barriers, immigration status, and limited formal education, many Latinx immigrants seek labor in agriculture. These farmworkers reside in some of the most remote locations in the US. where farmlands abound and labor under unforgiving conditions to meet production demands and feed their families. Their labor makes substantial contributions to both international food security and the U.S. economy. However, their labor comes at a cost. Participants in our study identified health concerns and illnesses linked to agricultural labor as well as the local environment.

Our findings on work-related illnesses, physical injury, musculoskeletal and skin disease, respiratory problems, and mental illness, echo the work of others [44,45,46,47,48,49]. We also found participants were concerned about illnesses associated with agricultural chemicals and pesticides, especially regarding children’s developmental health. With the increased use of agricultural chemicals, Latinx farmworkers experience levels of lifetime and residential pesticide exposure significantly greater than non-farmworkers [50]. Farmworkers exposed to agricultural chemicals carry them into their homes through soiled clothing, shoes, or contaminated skin, exposing non-farm working residents in the home (e.g., children) to chemicals that persist indoors [51]. Despite the growing number of studies showing the role of pesticides in child development, those who are most at risk (e.g., Latinx farm-working communities) lack information on agricultural chemical health risks [52].

Such working conditions require farmworkers, supervisors, and employers to practice and promote workplace safety not only for on-the-job risk reduction but also to reduce the effects of chemical exposure for family and community. For the participants in our study, reducing exposure to chemicals was difficult either because they had little to no training or had no way to remove themselves from chemical exposure. For instance, with limited transportation and employment options, most of our participants resided near or within farmlands and were continuously exposed to pesticides. While agricultural workers are required to take pesticide training as they are mandated by the US Environmental Protection Agency through the Worker Protection Standard, national data suggest only slightly more than half (57%) of farmworkers report having received pesticide training (NAWS 2015–2016).

In addition to concerns around pesticide exposure, participants discussed an increase in respiratory illnesses among adults and children. Proximity to the Salton Sea, a desiccating body of water that has been used to drain agricultural runoff from the nearby farmlands creating a toxic body of water, was commonly indicated as a contributing factor. Many participants expressed concern about the Sea’s smells, its toxic dust, and the harm it causes to human health, especially chronic child health conditions (e.g., asthma, bronchitis). Apprehensions about the Salton Sea and its effect on community health match the research community’s understanding of the release of the Sea’s toxicities in the form of water and dust, the latter of which is carried into homes and community spaces through windstorms [53]. This toxic dust is likely the cause of the ECV’s disproportionate pediatric population living with asthma and higher rates of asthma-related emergency visits compared to national averages [54].

## 5. Limitations and Next Steps

Study findings offer important insight into perceptions of common health concerns among Latinx farm working communities in the ECV, as well as barriers to accessing healthcare services and public health information. Yet, several limitations should be considered when interpreting the findings. First, study participant characteristics are similar to those of the general population characteristics of communities that border the northern part of the Salton Sea but also differ in important ways [55]. According to the 2021 US Census, 100% of the population in Mecca, California reported their ethnic identity as Latino/Hispanic and 97.5% reported speaking a language other than English at home, which mirrors our sample. However, the study sample compared to the general population characteristics of the area was slightly more educated, 34% of the sample had a high school degree/GED or higher versus 28.1% of the population, more representative of women’s perspectives (74% women in sample vs. 42.2% in the general population), older than the general population (roughly a 1/3 were over 50 years of age), and more likely to have healthcare insurance (43% vs. 11.5% in the general population). However, the number of study participants with healthcare insurance is similar to national estimates of farm workers indicating about half (47%) have health insurance (NAWS 2015–2016) with approximately 22% being insured by Medicaid or Medicare and 29% by employer. These demographic differences suggest that more vulnerable community members such as those without healthcare insurance and limited formal education were less represented in our study.

Furthermore, it was difficult to recruit men for our study and children between the ages of 0 to 18 were not included in the in-home meetings. Thus, the results more so reflect the experiences and perspectives of adult women (mothers, wives, young women) in farm working communities. Last, the original study, which was exploratory and intended to elicit community health concerns did not set out to focus on workplace health or environmental health hazards. However, during the analysis workplace health, specifically in the context of agricultural labor, and the health effects of the Salton Sea both emerged as important themes and were further developed.

Despite these limitations, this work laid the foundation for subsequent research and healthcare service delivery by creating a baseline understanding of community health needs. The main findings, published in Migration and Health, informed a policy brief published in English and Spanish that served as the basis for several lines of research including child health specifically childhood asthma and childhood obesity, as well as adult obesity and diabetes prevention, and mental health [21]. We have also developed innovative healthcare delivery models involving mobile units, pop-up clinics, and *promotoras* in reducing barriers to healthcare access [20]. Furthermore, all of our research, healthcare service delivery, and public health dissemination are in English, Spanish, and Purépecha to increase access to in-language services. Such approaches respond to study participants’ concerns for increased access to accurate information in Spanish provided by well-trained physicians and *promotoras*—the latter of whom play a critical role in healthcare delivery as local physicians and medical facilities are scarce [56].

## 6. Conclusions

US agricultural production demands place a heavy burden on Latinx immigrant farmworkers shaping their health and often decisions about their living conditions. Farmworkers often live in isolated regions with limited local resources, including healthcare facilities to address the detrimental effects of their living and work environments on personal and family health. Often without health insurance, access to medical facilities, sick pay, transportation, and/or fluency in the English language, rural Latinx farmworkers are at a greater disadvantage than others when it comes to health care access. 

The US depends on Latinx immigrant human labor to meet the demands of agricultural production. The health of these workers and their families should be a national priority. While farm labor is inherently a risky job, risk-reduction efforts via timely, legally mandated workplace health hazard training, as well as public health information about the effects of local environments on health outcomes, are crucial to reducing chronic health conditions among families and children in Latinx farm working communities. Furthermore, national associations that survey farm working populations such as the National Agricultural Worker Survey should consider assessing not only workplace hazards but also environmental hazards on the health of farm working communities.

## Figures and Tables

**Table 1:** In-home meeting interview guide.

1. Within the five general health-related categories below, what health concerns, conditions, or illnesses affect your community? - Children’s health - Physical health - Mental health - Reproductive health - Substance Abuse
2. Among the five health categories, vote for the three most important.
3. Among each of the three most important categories, how could those health concerns be reduced?
4. Among each of the three most important categories, what are some barriers or challenges to accessing health services?
5. How could we facilitate access to those health services or make them more accessible?
6. Looking at a map of your community, mark where you consider to be the most ideal location for health services to be provided.

**Table 2:** Demographics and characteristics of participants (*n* = 76).

Demographics	*n* (%)
Ethnicity/Race	
Latino/Hispanic	72 (95)
Indigenous	1 (1)
Not indicated	3 (4)
Age	
18 to 24	7 (9)
25 to 39	20 (26)
40 to 49	22 (29)
50+	27 (36)
Gender	
Female	56 (74)
Male	20 (26)
Country of Origin	
México	68 (90)
Guatemala	1 (1)
El Salvador	1 (1)
Other (US)	1 (1)
Not indicated	5 (7)
Language	
Spanish-speaking only	62 (82)
Purépecha-speaking only	5 (7)
Bilingual English and Spanish	5 (7)
Marital Status	
Married or civil union	54 (71)
Single	11 (14)
Divorced or separated	6 (8)
Widowed	3 (4)
Single mother	1 (1)
Not indicated	2 (3)
Highest level of education:	
None	6 (6)
Kindergarten	3 (4)
Grade 1–8	37 (49)
Grades 9–11	9 (12)
Some college or technical school	11 (14)
College 4 years	3 (4)
Graduate school or advanced degree	3 (4)
Not indicated	2 (3)
Housing	
Trailer	44 (52)
House	24 (32)
Converted garage	3 (4)
Vehicle	2 (3)
Apartment	1 (1)
Not indicated	2 (3)
Employment	50 (66)
Current Employment as Farm worker	45 (59)
Healthcare Services	
Health insurance	33 (43)
Access to local healthcare clinic	39 (51)
Had visited a doctor in the last 12 months	48 (63)

## Data Availability

Not applicable.
